# The role of bronchial epithelial cells in the pathogenesis of COPD in Z-alpha-1 antitrypsin deficiency

**DOI:** 10.1186/s12931-014-0112-3

**Published:** 2014-09-14

**Authors:** Laura Pini, Laura Tiberio, Narayanan Venkatesan, Michela Bezzi, Luciano Corda, Maurizio Luisetti, Ilaria Ferrarotti, Mario Malerba, David A Lomas, Sabina Janciauskiene, Enrico Vizzardi, Denise Modina, Luisa Schiaffonati, Claudio Tantucci

**Affiliations:** Department of Clinical and Experimental Sciences, University of Brescia, Brescia, Italy; Department of Molecular and Translational Medicine, University of Brescia, Brescia, Italy; Meakins Christie Laboratories, McGill University, Montreal, Canada; Bronchoscopy Department of Spedali Civili di Brescia, Brescia, Italy; Internal Medicine Department of Spedali Civili di Brescia, Brescia, Italy; Department of Respiratory Medicine, Policlinico S. Matteo, University of Pavia, Pavia, Italy; Faculty of Medical Sciences, University College London, London, UK; Hannover Medical School, Hannover, Germany; Unit of Cardiologic Medicine, Department of Medical and Surgical Sciences, University of Brescia, Brescia, Italy

**Keywords:** Z-AAT polymers, Bronchial epithelial cells, Pathogenesis of COPD, Bronchial epithelial cell dysfunction

## Abstract

**Background:**

Alpha-1 antitrypsin is the main inhibitor of neutrophil elastase in the lung. Although it is principally synthesized by hepatocytes, alpha-1 antitrypsin is also secreted by bronchial epithelial cells. Gene mutations can lead to alpha-1 antitrypsin deficiency, with the Z variant being the most clinically relevant due to its propensity to polymerize. The ability of bronchial epithelial cells to produce Z-variant protein and its polymers is unknown.

We investigated the expression, accumulation, and secretion of Z-alpha-1 antitrypsin and its polymers in cultures of transfected cells and in cells originating from alpha-1 antitrypsin-deficient patients.

**Methods:**

Experiments using a conformation-specific antibody were carried out on M- and Z-variant–transfected 16HBE cells and on bronchial biopsies and *ex vivo* bronchial epithelial cells from Z and M homozygous patients. In addition, the effect of an inflammatory stimulus on Z-variant polymer formation, elicited by Oncostatin M, was investigated. Comparisons of groups were performed using *t*-test or ANOVA. Non-normally distributed data were assessed by Mann–Whitney U test or the Kruskal-Wallis test, where appropriate. A *P* value of < 0.05 was considered to be significant.

**Results:**

Alpha-1 antitrypsin polymers were found at a higher concentration in the culture medium of *ex vivo* bronchial epithelial cells from Z-variant homozygotes, compared with M-variant homozygotes (*P* < 0.01), and detected in the bronchial epithelial cells and submucosa of patient biopsies. Oncostatin M significantly increased the expression of alpha-1 antitrypsin mRNA and protein (*P* < 0.05), and the presence of Z-variant polymers in *ex vivo* cells (*P* < 0.01).

**Conclusions:**

Polymers of Z-alpha-1 antitrypsin form in bronchial epithelial cells, suggesting that these cells may be involved in the pathogenesis of lung emphysema and in bronchial epithelial cell dysfunction.

**Electronic supplementary material:**

The online version of this article (doi:10.1186/s12931-014-0112-3) contains supplementary material, which is available to authorized users.

## Background

Alpha-1 antitrypsin (AAT) is the main proteinase inhibitor within the lung. It is produced primarily by hepatocytes from where it is secreted into the plasma. It then diffuses into the lung where it acts as the main inhibitor of neutrophil elastase [1,2]. It is also produced by bronchial epithelial cells (BECs), macrophages, and polymorphonuclear leukocytes and neutrophils [3-5].

Wild-type AAT protein is known as M-AAT [6], however, more than 100 variants have been described of which the Z variant is the most clinically relevant. The Z mutant of AAT has a point mutation, Glu342Lys, in the hinge region of the molecule that renders it inactive and prone to intermolecular linkage and loop-sheet polymerization. Polymers of Z-AAT aggregate within the cells that produce this form of the protein leading to reduced AAT secretion.

The pathogenesis of emphysema in Z-AAT homozygotes is complex, but two main mechanisms have been reported. Firstly, severe deficiency of AAT within the alveoli allows uncontrolled proteolytic attack and tissue destruction. This mechanism has formed the cornerstone of the proteinase-antiproteinase hypothesis of tissue damage [7-14]. Secondly, Z-AAT is approximately five-fold less efficient at inhibiting neutrophil elastase than normal M-AAT [15,16]. An additional mechanism may be the pro-inflammatory effect of Z-AAT polymers in the lungs: polymers can be detected in Z-AAT homozygotes’ bronchoalveolar lavage fluid, they are chemotactic to neutrophils *in vivo* [17,18], they co-localize with neutrophils in the alveoli of Z-AAT patients, and they are pro-inflammatory in cell and mouse models of disease [19]. These data raised the additional hypothesis that Z-AAT undergoes a conformational transition to polymers within the lungs and that this transforms AAT into a local pro-inflammatory stimulus [17-20], which provides an explanation for the excessive number of neutrophils in the lungs of Z-AAT homozygotes and the progression of disease, despite adequate AAT replacement [19].

Mechanisms that drive formation of Z-AAT polymers in the lung and their cellular origin are still unknown. These polymers could be derived from circulating monomeric plasma AAT, from polymorphonuclear neutrophils, or from local respiratory cells. It is known that AAT can be synthesized and secreted by BECs, especially during inflammation, but little is known about the synthesis, accumulation, and secretion of Z-AAT or its polymers by BECs. In addition, the hypothetical cytotoxic effect (either direct or in association with the neutrophils) of polymer accumulation in airway epithelial cells has yet to be shown. To elucidate the source of Z-AAT polymers in the lung and the role of BECs in the pathogenesis of lung emphysema and airway dysfunction in AAT-deficient patients, we have investigated the expression, accumulation, and secretion of Z-AAT protein by BECs, with particular attention to the presence of Z-AAT polymers. In addition, the effect of an inflammatory stimulus on this process, provided by Oncostatin M, was analyzed to provide further insights as to whether inflammation exacerbates the formation of Z-AAT polymers.

## Methods

### Patient selection

Homozygous patients for Z-AAT and M-AAT (seven for each genotype) with diagnosed emphysema were selected at our Regional Reference Centre for AAT Deficiency (Department of Internal Medicine, Brescia, Italy) [21,22] following approval from ethics committees of Spedali Civili of Brescia and having obtained informed consent. At the time of inclusion, subjects were aged 18–70 years, non- or ex-smokers (>10 pack-years) for at least 5 years, and in a stable condition (Table 1). The exclusion criteria are detailed in the Additional file 1.

**Table 1:** **Patient characteristics**

	**M-AAT (n = 7)**	**Z-AAT (n = 7)**	***P*** **value**
Gender			
*Male*	6	4	-
*Female*	1	3	-
Age (yr) M ± SEM	64.2 ± 21	48 ± 6.70	0.4765
AAT (mg/dl) M ± SEM	130 ± 26	49.29 ± 14.18	0.0184
FEV_1_ (% predicted) M ± SEM	69.7 ± 28.1	77 ± 15.20	0.8231

*Definition of abbreviations*: AAT = alpha-1 antitrypsin; FEV_1_ = forced expiratory volume in 1 second; M = mean; SEM = standard error of mean.

A complete medical history was collected, with particular attention to previous smoking and pharmacologic therapy. Plasma AAT levels were measured and the patient’s genotype determined by polymerase chain reaction (PCR) with fluorescently labelled Taq-Man probes (Vic or Fam labels) on a LightCycler480 (Roche Diagnostics Limited, Burgess Hill, UK). High-resolution computed tomography was performed to detect the extent of pulmonary emphysema. Arterial blood gas analysis and pulmonary function tests, including spirometry, flow-volume curve, lung volume measurements, and carbon monoxide gas-transfer factor, were performed.

### Bronchoscopy

Bronchoscopy was performed according to standard procedures (please refer to Additional file 1). Two to four biopsy specimens per patient were taken from the carina of segmental bronchi using cup forceps. Biopsy specimens were placed in Tris-buffered saline solution and immediately processed for immunohistochemical experiments. Tissues were fixed in 4% v/v paraformaldehyde, embedded in paraffin, sectioned (2 μm), placed on glass slides coated with poly-L-lysine (0.1% w/v), and baked overnight at 37°C.

Epithelial brushings were obtained using a fiber-optic bronchoscope in accordance with standard guidelines [23]. BECs were obtained using a sterile single-sheathed nylon cytology brush. On average, seven to eight consecutive brushings were sampled from the bronchial mucosa of the second- and third-generation bronchi. Cells were harvested into 5-ml sterile phosphate-buffered saline after each brushing, and then recovered by centrifugation [24].

### 16HBE cell culture and transfections

The human BECs (16HBE) [25] were maintained in culture as described [26]. Complementary DNA for human M and variant Z (G342L) AAT cloned into the mammalian expression vector pcDNA3.1/Zeo (+) were transiently transfected into cells using Lipofectamine™ 2000 (Invitrogen, Carlsbad, CA, USA), according to the manufacturer’s instructions. For further detail please refer to Additional file 1.

### Immunohistochemistry of biopsy tissue

Tissue biopsy were obtained according to standard procedures as detailed in the Additional file 1. To detect positive staining of the epithelial layer, paraformaldehyde-fixed, paraffin-embedded biopsy sections were incubated with the ATZ11 antibody, which binds with high affinity to polymeric Z-AAT [27], or the isotype control mouse IgG (Vector Laboratories, Orton Southgate, Peterborough, UK). Sections were developed with Fast Red salt and counterstained with Gill II hematoxylin. Two blinded observers per patient group analyzed and quantified the tissue distribution of AAT. For further information, please refer to Additional file 1.

### *Ex vivo* cell cultures

Primary cultures of cells from bronchial epithelial cells were established as described previously [24]. Minor modifications are detailed in the Additional file 1.

### Oncostatin M treatment

Cultures of untransfected 16HBE cells and *ex vivo* primary cultures of human BECs were supplemented with 50 ng/ml Oncostatin M (R&D Systems Inc., Minneapolis, MN, USA) for 24 hours.

### Western blots to assess AAT expression

Sodium dodecyl sulphate (SDS) or nondenaturing polyacrylamide gel electrophoresis (PAGE) followed by Western blot analyses were carried out on transfected and nontransfected 16HBE cells and *ex vivo* cultured BECs, using an anti-AAT antibody that detect all conformations of AAT (Total-AAT, DakoCytomation Ltd) or ATZ11 antibody.

### Quantification of AAT expression

Reverse transcription real-time PCR (real-time-PCR) and enzyme-linked immunosorbent assay (ELISA) experiments were used to detect respectively the expression levels of AAT mRNA and the protein levels of monomeric and polymeric AAT from 16HBE cells and BECs. For detailed methods of these experiments, please refer to the Additional file 1.

### Statistical analysis

Statistical analysis was performed with the SPSS software package (SPSS, Chicago, IL, USA). Comparisons of groups were performed using *t*-test or ANOVA. Non-normally distributed data were assessed by Mann–Whitney U test or the Kruskal-Wallis test, where appropriate. A *P* value of < 0.05 was considered to be significant.

## Results

### Expression of M-AAT and Z-AAT by transfected 16HBE cells

**Figure 1:**
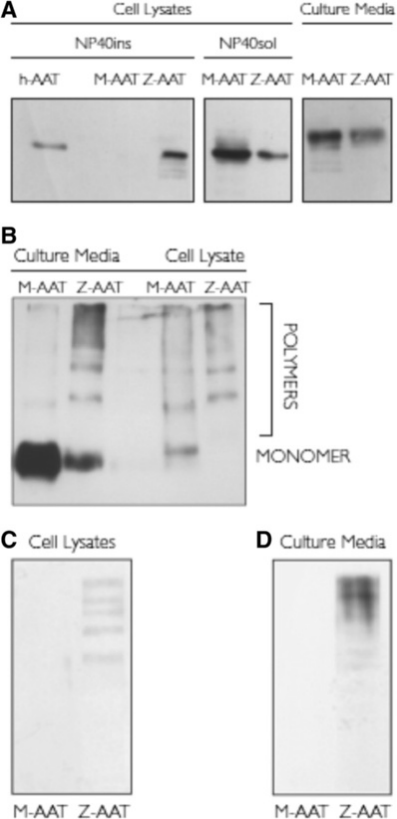
**Expression of M- or Z-AAT in the 16HBE cell line. (** ***A*** **)** 16HBE cells expressing M- or Z-AAT were assayed for AAT expression by SDS PAGE and Western blot analysis. 24 hours after transfection, proteins from 50 μl of cell culture media or from 10 μg or 10 μl of NP40-soluble and insoluble cell lysates respectively were separated by SDS-PAGE, transferred to PVDF membranes, and hybridized with anti-AAT antibody to detect total AAT protein. Purified human serum AAT (hAAT) was used as positive control. Typical fluorograms from three independent experiments, which gave superimposable results, are shown. **(** ***B*** **)** Nondenaturing PAGE and Western blot analysis using anti-AAT antibody to detect total AAT in 10 μg of NP40-soluble cell lysate and 10 μl of cell culture media from 16HBE cells expressing M- or Z-AAT. The migration of polymeric and monomeric AAT species is indicated. Typical fluorograms from three independent experiments, which gave superimposable results, are shown. **(** ***C*** **)** Nondenaturing PAGE and Western blot analysis using the antipolymeric Z-AAT antibody ATZ11 in NP40-soluble cell lysate fraction (10 μg) and **(** ***D*** **)** cell culture media (50 μl)from 16HBE cells expressing M- or Z-AAT. Typical fluorograms from three independent experiments, which gave superimposable results, are shown. *Definition of abbreviations*: AAT = alpha-1 antitrypsin; h-AAT = human serum AAT; PAGE = polyacrylamide gel electrophoresis; PVDF = polyvinylidene fluoride; SDS-PAGE = sodium dodecyl sulphate polyacrylamide gel electrophoresis.

Initial experiments were undertaken in the immortalized BEC line (16HBE), which was engineered to express human Z and M-AAT. SDS-PAGE and Western blot analysis of the transfected 16HBE cell line demonstrated that AAT was only detected in the NP40-insoluble fraction of lysates from cells transfected with Z-AAT, suggesting that only the mutant protein forms polymers, and that a fraction of these are insoluble. In both the NP40-soluble fraction of the cell lysate and in the culture medium, a higher amount of AAT was detected for M-expressing 16HBE cells, when compared to Z-expressing 16HBE cells (Figure 1A).

### Polymers of Z-AAT form within transfected 16HBE cells

To further characterize the AAT protein produced by transfected cells, we used a nondenaturing PAGE protocol to resolve any conformers of the protein that might be produced. Western blot analyses using a polyclonal antibody that detects all AAT revealed that both in the medium and the NP40-soluble cell lysate from M-AAT transfected cells, AAT protein was primarily in the monomeric form. This contrasted with the protein that was detected in Z-AAT transfected cells, which showed a high proportion of polymers in both the cell lysate and the culture medium (Figure 1B). To further investigate the production of AAT polymers, we performed nondenaturing PAGE and Western blot analysis on the NP40-soluble fraction of the cell lysate and culture media from transfected 16HBE cells using the ATZ11 antibody. Polymers were not detected in cells that had been transfected with M-AAT, which contrasted with the presence of AAT polymers in Z-transfectants (Figures 1C and D).

### Inflammation increases the endogenous expression of M-AAT in 16HBE cells

**Figure 2:**
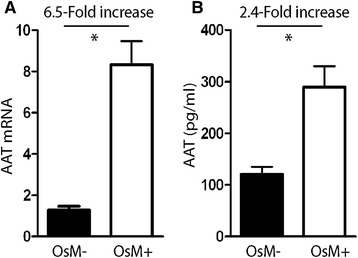
**Effects of Oncostatin M on expression of endogenous AAT in the 16HBE cell line.** Nontransfected 16HBE cells were cultured in the absence (OsM-) or presence (OsM+) of 50 ng/ml Oncostatin M for 24 hours, and AAT mRNA expression and AAT protein secretion were analyzed. **(** ***A*** **)** Mean AAT mRNA expression levels, normalized for β-actin expression levels (×10^3^), as measured by real-time PCR analysis from total cellular RNA. **(** ***B*** **)** AAT protein levels as measured by ELISA for total AAT in cell culture media. The fold increase in expression of Oncostatin M-treated versus untreated cells is indicated. Results are expressed as mean ± SEM (n = 3–4). *P < 0.05 Student’s *t*-test. *Definition of abbreviations*: AAT = alpha-1 antitrypsin; ELISA = enzyme-linked immunosorbent assay; mRNA = messenger ribonucleic acid; Real-time PCR = reverse transcription real-time polymerase chain reaction; RNA = ribonucleic acid.

The effect of an inflammatory stimulus, provided by Oncostatin M, on endogenous expression of AAT by 16HBE cells was assessed by real-time PCR and ELISA following exposure to Oncostatin M for 24 hours. Results from real-time PCR, carried out on total ribonucleic acid (RNA) extracted from cell lysates, demonstrated that expression of AAT increased 6.5-fold in Oncostatin M-treated cells, as compared to nontreated cells (*P* < 0.05; Figure 2A). ELISA carried out on the cell culture medium detected a 2.4-fold increase of AAT protein in Oncostatin M-treated versus nontreated cells (*P* < 0.05; Figure 2B).

### Bronchial biopsies from Z-AAT homozygous patients contain polymers

**Figure 3:**
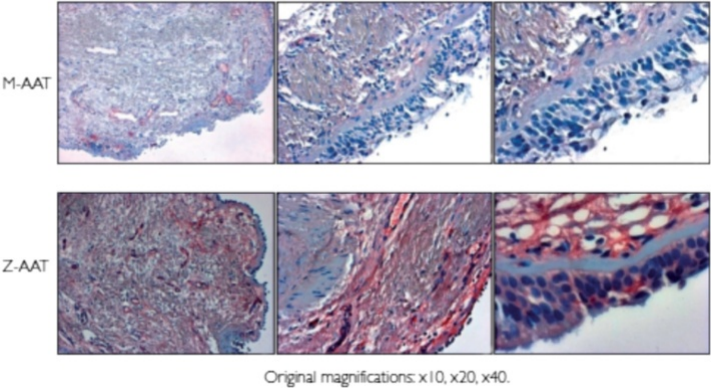
**Immunohistochemistry of bronchial epithelial biopsy.** Endobronchial biopsy sections taken from patients homozygous for M-AAT or Z-AAT were fixed and stained with the ATZ11 antibody. Evidence of polymers of Z-AAT was confirmed in the epithelial cells and submucosa of Z-AAT homozygote tissue. *Definition of abbreviation*: AAT = alpha-1 antitrypsin.

Immunohistochemistry was performed on bronchial biopsies. Staining with the ATZ11 antibody revealed that biopsies from M-AAT homozygous patients showed no evidence of AAT in non-native conformations (Figure 3). In contrast, positive staining was observed in the submucosa and epithelial cells (Figure 3) of Z-AAT homozygotes, suggesting the presence of Z-AAT polymers. As previously reported [26], positive staining was also observed in endothelial vessels. The specificity of immunostaining was demonstrated by the absence of a signal in sections incubated with the antibody isotype control and by omission of the primary antibody (data not shown).

### AAT is normally produced by BECs cultured *ex vivo*

**Figure 4:**
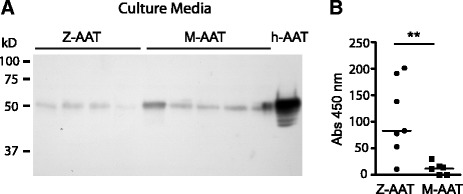
**Detection of AAT in ex-vivo cultured BECs. (** ***A*** **)** SDS-PAGE and Western blot analysis using an antibody to detect total AAT in 20 μl of 10x concentrated culture media from a 72-hour culture of *ex vivo* BECs from five patients homozygous for M-AAT and four patients homozygous for Z-AAT. Purified human serum AAT (h-AAT) served as positive control. A typical fluorogram from three independent experiments, which gave superimposable results, is shown. **(** ***B*** **)** Cell culture media from a 72-hour culture of *ex vivo* BECs from six patients homozygous for M-AAT and seven patients homozygous for Z-AAT were concentrated 10 times, opportunely diluted to a final concentration of 10 μg/ml of AAT as assessed by an ELISA for total AAT, and were analyzed by ELISA using the antipolymeric AAT antibody ATZ11. . * *P* = 0.01 Mann–Whitney U test. *Definition of abbreviations*: AAT = alpha-1 antitrypsin; BEC = bronchial epithelial cell; h-AAT = human serum AAT; PAGE = polyacrylamide gel electrophoresis; ELISA = enzyme-linked immunosorbent assay.

Secretion of AAT by BECs collected from M- and Z-AAT homozygous patients was investigated. SDS-PAGE followed by Western blot analyses with a polyclonal anti-AAT antibody was performed on the culture media of *ex vivo* cultured cells that had been collected by epithelial brushing. Consistent with the results obtained from transfected 16HBE cells, epithelial cells derived from Z-AAT patients were able to secrete AAT protein, but to a lesser degree than cells derived from M-AAT patients (Figure 4A). The AAT protein present in the medium for both types of cells was in the form of a single band running at the same position as a purified human AAT control. There was no evidence of complexed or cleaved AAT in these samples.

### Z-AAT from BECs cultured *ex vivo* forms polymers

ELISA was performed on the culture media of BECs from M- and Z-AAT homozygotes using the ATZ11 antibody. We found a significantly higher signal in the samples from Z-AAT patients compared with those from M-AAT patients (*P* < 0.01; Figure 4B). These results are consistent with the presence of Z-AAT polymers in culture medium conditioned by Z-AAT BECs, in agreement with our findings in transfected cultured cells.

### Inflammation increases the expression of AAT and the formation of Z-AAT polymers in BECs cultured *ex vivo*

**Figure 5:**
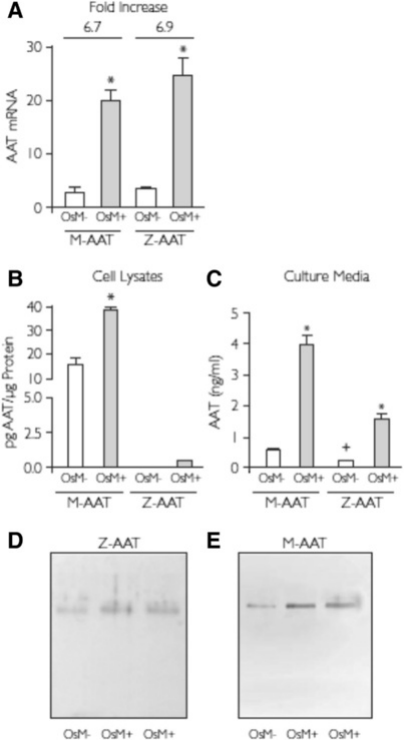
**The effect of Oncostatin M on Z-AAT expression in ex-vivo cultured BECs. (** ***A*** **)** Real-time PCR analysis for AAT mRNA in *ex vivo* cultured BECs from patients homozygous for M-AAT or Z-AAT. Cells from four patients homozygous for Z-AAT and five patients homozygous for M-AAT were incubated in the absence (OsM-) or presence (OsM+) of Oncostatin M (50 ng/ml) for 24 hours. After treatment cells from M-AAT and Z-AAT patients were pooled and AAT mRNA levels were measured. The fold increase in expression of Oncostatin M-treated versus control cells is indicated. Results are expressed as mean ± SEM (n = 3–4). *P < 0.05 Student’s *t*-test. **(** ***B*** **)** ELISA for total AAT protein in cell lysates and **(** ***C*** **)** cell culture media of *ex vivo* cultured BECs from four Z-AAT and five M-AAT homozygous patients. Cells were incubated in the absence (OsM-) or presence (OsM+) of Oncostatin M (50 ng/ml) for 24 hours and the intracellular AAT accumulation and secretion were measured for lysates of pooled cells and 10x concentrated cell culture media, respectively. Results are expressed as mean ± SEM (n = 3–4). *P < 0.05 ANOVA followed by Bonferroni *t*-test, Oncostatin M-treated versus untreated cells; +*P* < 0.05 ANOVA followed by Bonferroni *t*-test, M-AAT– versus Z-AAT–expressing cells. SDS-PAGE and Western blot analysis using an antibody to detect total AAT was carried out on the cell culture media of *ex vivo* cultured BECs. Cells from five patients homozygous for Z-AAT **(** ***D*** **)** and five patients homozygous for M-AAT **(** ***E*** **)** were cultured in the absence (OsM-) or presence (OsM+) of 50 ng/ml of Oncostatin M. At 24 hours after treatment cell media were harvested and concentrated 10x. Typical fluorograms from three independent experiments, which gave superimposable results, are shown.

To investigate whether AAT expression by patient-derived BECs was increased by inflammation, cell cultures were supplemented with Oncostatin M. BECs from homozygous M-AAT or Z-AAT patients were exposed to Oncostatin M for 24 hours or left untreated, and cellular levels of AAT messenger RNA (mRNA) were analyzed by real-time PCR. There was a significant increase in levels of AAT mRNA in cells exposed to Oncostatin M compared to untreated cells (*P* < 0.05), which was detected in both M- and Z-AAT–expressing cells, at a comparable level (6.7- vs. 6.9-fold increase, respectively; Figure 5A).

To confirm that AAT mRNA induction by Oncostatin M treatment of BECs actually leads to an increase in AAT protein accumulation and secretion, an ELISA using the antibody for total AAT was carried out on cell lysates and culture media of cells from M- and Z-AAT homozygotes. Cells from different patients of each genotype were exposed to Oncostatin M for 24 hours or left untreated. In the lysate of M-AAT–expressing cells, a significant increase in AAT protein was present in Oncostatin M-treated cells compared with the untreated cells (*P* < 0.05), whereas, in Z-AAT cell lysates, AAT basal level was undetectable and became measurable only after Oncostatin M stimulation (Figure 5B). In culture media from untreated cells, a higher concentration of AAT was present from M-AAT when compared to Z-AAT–expressing cells (*P* < 0.05, Figure 5C). Oncostatin M treatment significantly increased the amount of AAT secreted in the media for both cell types (*P* < 0.05). It is noteworthy that, irrespective of the total AAT amount, Oncostatin M treatment induces the same fold increase of secreted AAT compared to basal levels of the protein level in the media from both M- and Z-AAT–expressing cells. The increased amount of AAT secreted in cell media from BECs treated with Oncostatin M was also seen when the samples were analyzed by SDS-PAGE and Western blot using a polyclonal anti-total AAT antibody (Figures 5D and E), in agreement with our ELISA results.

**Figure 6:**
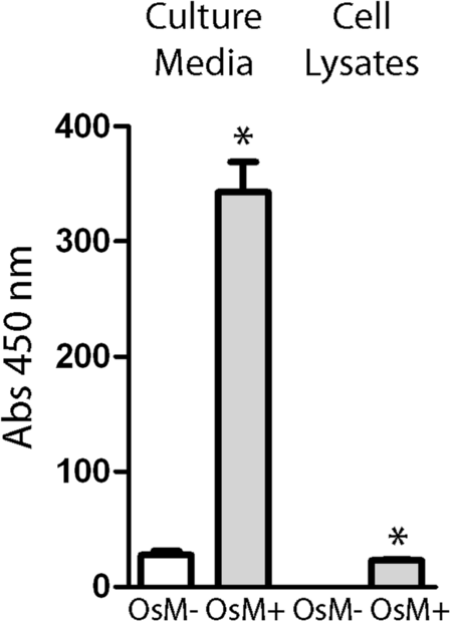
**Effect of OsM on AAT polymers formation in ex-vivo cultured BECs.** ELISA for polymeric AAT protein, using the ATZ11 antibody, in cell culture media and cell lysates of *ex vivo* cultured BECs from Z-AAT homozygous patients. Cells from five patients were incubated in the absence (OsM-) and presence (OsM+) of Oncostatin M (50 ng/ml) for 24 hours, and AAT secretion and intracellular accumulation were measured in the 10x concentrated cell medium. Results are expressed as mean absorbance ± SEM (n = 3–4), measured at 450 nm. *P = < 0.01 Student’s *t*-test. *Definition of abbreviations*: AAT = alpha-1 antitrypsin; BEC = bronchial epithelial cell; ELISA = enzyme-linked immunosorbent assay; SEM = standard error of mean.

To analyse the effect of Oncostatin M treatment on AAT polymer accumulation and secretion in BECs, we performed ELISA with the ATZ11 antibody on pooled samples of patients’ Z-AAT bronchial cells cultured in the presence of Oncostatin M for 24 hours or left untreated. Oncostatin M significantly increased the ATZ11 signal in cell culture medium and within the BECs (*P* < 0.01), consistent with an increase in Z-AAT polymer accumulation and secretion during inflammatory conditions (Figure 6).

## Discussion

Here we provide, for the first time, strong evidence that BECs derived from Z-AAT patients are able to accumulate and secrete Z-AAT in the form of polymers. Moreover, we show that secretion of Z-AAT is increased in response to inflammation. Initially, we undertook studies in 16HBE cells engineered to express M- or Z-AAT to detect the expression of AAT protein, and to establish the degree to which Z-AAT and M-AAT were expressed, retained, or secreted in this *in vitro* model. The rationale for using this cell line was based on previous observations of these cells’ ability to retain the morphology and function of normal human airway epithelial cells [25]. Our results reveal that Z but not M-AAT is present in the NP40-insoluble cell fraction of cell lysates, likely due to the formation of Z-AAT polymers in these cells. This could lead to the formation of Z-protein globular inclusions within the cells, in a mechanism analogous to that observed in hepatocytes [28]. Our analysis also showed that a higher amount of soluble intracellular AAT is present in M- rather than Z-AAT–expressing cells. These data, which agree with results from a previous study performed using Chinese Hamster Ovary cells [29], may be explained by increased degradation of the mutant protein and by its sequestration as aggregates within the insoluble fraction. AAT protein is efficiently secreted into the cell media by M-AAT–expressing cells, while only a lower amount of the protein was found in the cell media from Z-AAT–expressing cells, as previously reported in other cell models of AAT deficiency [29-31].

The results obtained in cell lines were consistent with our observations from BECs originating from patients who were homozygous for M- or Z-AAT. We have shown that BECs from Z-AAT homozygous patients secrete less AAT than those from M-AAT homozygous patients. Our data also indicate that these differences do not arise from differences at the transcriptional level, since a comparable amount of AAT mRNA was detected in the cells from both sets of patients. This suggests that, similar to what we describe in our *in vitro* cell model, and as widely reported in the literature for different cell types, low levels of Z-AAT protein in *ex vivo* BEC cultures can be ascribed to post-translational events, including intracellular degradation and/or accumulation as intracellular polymers.

Aberrantly polymerized Z-AAT protein is known to accumulate in the endoplasmic reticulum (ER) of hepatocytes [28]. Accumulation of these polymers within the ER can induce or predispose to the activation of cellular stress responses, including the unfolded protein response and the ER overload response [29,31,32]. In hepatocytes, Z-AAT accumulation can lead to apoptosis whereas, paradoxically, it is associated with cell survival in BECs [31]. The pathophysiological effect of Z-AAT is thus manifold: the secretion of functional circulating AAT is markedly decreased, creating a protease/antiprotease imbalance with neutrophil elastase; accumulation of Z-AAT polymers can have a deleterious effect on the ER, leading to the activation of ER stress pathways; and, depending on the producer cell, accumulation of Z-AAT can mediate either cell survival or apoptosis.

The combined processes of intracellular degradation and retention of Z-AAT contribute to the pathogenesis of AAT deficiency, however, it has been proposed that the pro-inflammatory effect of secreted Z-AAT polymers could exacerbate the tissue damage that occurs in emphysema [33]. It is thus important to investigate the presence of Z-AAT polymers produced locally by lung epithelial cells, in addition to circulating polymers that can arise from the liver. This was assessed with a conformation-specific monoclonal antibody, the ATZ11 antibody, which has been used in the past to detect polymers of Z-AAT [19,27].

Here we demonstrate the presence of intracellular and secreted Z-AAT polymer in Z-AAT–expressing 16HBE cells. The small proportion of M polymer that was also detected in cell lysates was unexpected and it is likely to be a result of the high expression of plasmid-derived M-AAT. Most importantly, ATZ11-positive Z-AAT was detected in bronchial biopsies from Z-AAT homozygous patients and in the cell media from their *ex vivo* BECs. This suggests a role for bronchial epithelium in Z-AAT polymer deposition in bronchial airways. It should be noted that, although we were able to detect Z-AAT polymers by native PAGE/WB in culture media from Z-AAT expressing 16HBE cells, definitive demonstration of the presence of Z-AAT polymers in culture media from patient’s cells was obtained only by using ELISA with ATZ11 antibodies, likely because the higher sensitivity of ELISA in comparison to native PAGE/WB technique. Results from *ex vivo* cells also suggest that the amount of polymeric Z-AAT that is produced by BECs could differ between individuals, as demonstrated by the variability that was observed in total Z-AAT secretion into culture media by patients’ cells. With this result in mind, it can be speculated that differing amounts of Z-AAT polymers could correlate with the degree of lung disease that is seen in AAT-deficient patients. Our results differ from the ones of Van’t Wout et co-authors [34] who did not find any evidence of Z-AAT polymers in cultured BEC from Z-AAT homozygous patients. This meaningful difference may be explained by an increased sensitivity of our experimental approach to detect Z-AAT polymers and by differences between the two experimental models. Indeed, differently from Van’t Wout and co-authors, we concentrated the sample before the analysis and used the anti-polymeric AAT antibodies ATZ11 which, although less specific, is more sensitive for Z-AAT polymers than the antibody they used [35]. We are also confident that signal we measured is due to the presence of polymers since we could not detect SDS-stable protease-AAT complex or cleaved AAT bands in our Western blots of culture medium from Z-AAT BECs, thus excluding the most relevant sources of potential false-positive ATZ11 signals. Moreover, in our experimental condition, BECs from both M-AAT and Z-AAT patients’ cells seem to produce higher amounts of AAT since, differently from what reported by Van’t Wout and co-authors, AAT production (both in cell lysate and culture media) is detectable already in basal condition thus allowing us to detect low but significant levels of polymeric Z-AAT.

The effect of inflammation on the expression of Z-AAT was also investigated, to ascertain whether it increases the expression of the mutant protein, as in the case of lung-derived epithelial cell expression of wild-type AAT [3]. Results from real-time PCR, denaturing SDS-PAGE, and ELISA showed that Oncostatin M treatment of patients’ BECs increases the expression of Z-AAT. Moreover, we demonstrate that the inflammatory stimulus increases the presence of Z-AAT polymers in the culture medium. Due to the pro-inflammatory and chemotactic function of AAT polymers [19], our findings may help to understand why the clinical outcome is so much worse for Z-AAT patients exposed to inflammatory agents such as cigarette smoke. Cigarette smoke may increase AAT production by BECs, leading to an increased deposition of polymeric AAT in Z-AAT individuals who smoke.

The accumulation of AAT polymers in BECs exposed to an inflammatory stimulus (Oncostatin M) may help us to understand why we did not detect polymeric AAT in cell lysates of primary cell cultures of untreated *ex vivo* bronchial cells, contrasting with observations from bronchial biopsies and transfected 16HBE cells. Tissue culture of *ex vivo* cells requires several proliferative cycles before a stable culture has been established, which could dilute pre-existing intracellular aggregates; moreover, cells in culture have a limited lifespan, which may explain why polymer accumulation might not be detectable at steady state conditions. Our results show that, upon treatment with Oncostatin M, the higher level of Z-AAT expression leads to increased deposition of intracellular Z-AAT polymers.

## Conclusion

In conclusion, our study shows that BECs from Z-AAT patients are able to express, accumulate, and secrete Z-AAT protein and its polymers, and that this process is increased by inflammatory stimuli. This suggests that these cells may be involved in the pathogenesis of lung emphysema and in BEC dysfunction. These findings strengthen the rationale for the development of new therapeutic approaches, such as treatments to prevent polymer formation, for managing AAT-deficiency–related diseases [36].

## Additional file

Additional file 1
**Supplemental Material and Methods [** 37,26,38 **-** 40 **].**

## Abbreviations

BECs: Bronchial Epithelial Cells
AAT: Alpha-1 antitrypsin
M-AAT: Wild-type AAT protein
Z-AAT: Z mutant of AAT
PCR: Polymerase Chain Reaction
16HBE: 16 Human Brochial Epithelial Cell line
Real-time-PCR: Reverse transcription real-time PCR
ELISA: Enzyme-linked immunosorbent assay
PAGE: Polyacrylamide gel electrophoresis
SDS: Sodium dodecyl sulphate
RNA: Ribonucleic acid
mRNA: Messenger RNA
ER: Endoplasmic reticulum
eALTA: European Alpha-1 Antitrypsin Laurell’s Training Award
