# Supplementary material for: The role of bronchial epithelial cells in the pathogenesis of COPD in Z-alpha-1 antitrypsin deficiency
Source: Respir Res. 2014 Sep 14;15(1):112. doi: 10.1186/s12931-014-0112-3 (PMC4177581; doi:10.1186/s12931-014-0112-3)
Supplement: Additional file 1 — Supplemental Material and Methods [ 37,26,38 - 40 ]. [file 12931_2014_112_MOESM1_ESM.doc]

**Additional file 1**

**METHODS**

**16HBE Cell Transfection**

Each well of a 6-well plate was transfected with 4 μg of mammalian vectors encoding human M- or Z-AAT [37] generously provided by Dr. Sifers (Houston, TX). Transfection efficiencies were assessed 24 hours after transfection by immunohistochemistry. Cells were grown on coverslips and then fixed with 4% paraformaldehyde. Following cell permeabilization and blocking of nonspecific antibody binding sites, cells were incubated with a polyclonal rabbit anti-human AAT (total AAT; DakoCytomation Ltd), which would recognize both monomeric and polymeric AAT forms, for 2 hours at room temperature. Immunocomplexes were then labelled with biotin-conjugated anti-rabbit IgG (Vector Laboratories) and visualized by the VECTASTAIN® ABC Kit (Vector Laboratories) and 3,3'-diaminobenzidine staining. Cells were counterstained by Mayer’s hematoxylin and examined by light microscopy.

24 hours after transfection, cell medium was collected and cells were lysed in lysis buffer containing 50 mM Tris-HCl (pH 7.4), 150 mM NaCl, 1% NP40, and protease inhibitors (Sigma, St Louis, MO, USA). Cell lysate was further fractioned by centrifugation at 12,000 g in NP40-soluble and insoluble fractions found in the supernatant and the pellet, respectively. The insoluble material recovered in the pellet was solubilized by boiling in 50 mM Tris/HCl (pH 6.5), 5% SDS, 10% glycerol and diluted 1:20.

**Patient Selection**

Exclusion criteria were smoker status (current or in the previous 5 years), history of exposure to any significant occupational factor and of passive smoke, recent upper respiratory infections, recent chronic obstructive lung disease exacerbation, initiation of systemic corticosteroid therapy, inhaled corticosteroids or antibiotics within the month preceding the study, bronchiectasis, chronic systemic diseases not related to AAT deficiency, breastfeeding, arrhythmias, anticoagulation therapy, coagulation diseases, and thrombocytopenia.

**Tissue Biopsy and Antibody Staining**

Patients were pretreated with 2.5 mg of nebulized salbutamol followed by intravenous fentanyl 50 mg and midazolam 2–10 mg until conscious sedation was achieved. Xylocaine was sprayed at the posterior pharynx and a flexible fiber-optic bronchoscope (Olympus BF Type XT20, Olympus, Southend-on-Sea, Essex, UK) was introduced via the mouth. 2-ml aliquots of 2% lignocaine were introduced via the bronchoscope and applied to the vocal cords, trachea, and left and right main stem bronchi. Endobronchial biopsies were then obtained from the subcarinae of the second- to fourth-generation bronchi of the right upper, right middle, and right lower lobe bronchi using BARD precisor pulmonary coated disposable biopsy forceps. Biopsy specimens were fixed in 4% paraformaldehyde and embedded in paraffin. Sections were blocked with ready-to-use serum-free protein block (DakoCytomation Ltd) and incubated overnight at 4°C with a 1:50 dilution of the primary monoclonal mouse anti-AAT polymer antibody ATZ11 (kindly provided by Dr. S. Janciauskiene, Hannover Medical School, Germany), which specifically binds to polymeric AAT [26]. The primary antibody was replaced by isotype-matched control antibody or buffer for negative controls. Slides were then incubated with biotinylated secondary mouse anti-IgG antibody followed by alkaline phosphatase-conjugated avidin for 1 hour. After further washing, immunolabelling was performed using an alkaline phosphatase anti-alkaline phosphatase method. Sections were developed with Fast Red Salt, after blocking endogenous alkaline phosphatase activity, and counterstained with Carazzi’s hematoxylin [38, 39]. Tissue sections were observed under an Olympus light microscope (Carson Group, Markam, ON, Canada) at a 40 × magnification. Digital images were captured using a high-definition digital camera attached to the microscope.

***Ex Vivo* Cell Cultures and Lysis**

Briefly, freshly-brushed BECs were seeded into culture dishes precoated with a mixture of type I collagen (Sigma), fibronectin (Sigma), and albumin (Sigma) in a serum-free hormonally supplemented Bronchial Epithelium Growth Medium (Clonetics, Basel, Switzerland) containing 50 µg/ml penicillin, 50 µg/ml streptomycin, and 50 µg/ml gentamycin. When confluent, cells were detached using trypsin and were allowed to further expand until used for experimentation at passage 2 or 3. Viability was assessed by exclusion of trypan blue dye and the epithelial nature of cells assessed by immunocytochemistry using a pan-CK antibody (Sigma) following manufacturers’ protocols. Culture media from confluent monolayers of primary culture of bronchial cells (2 × 10^4^/cm^2^ cells) were collected at 24 and 72 hours after seeding, added with a protease inhibitor cocktail (Sigma), centrifuged and concentrated 10 × by using Vivaspin 2 ultrafiltration spin columns (10,000 MW cut-off; Sartorius Biolabs, Firenze, Italy) following manufacturers’ instructions. Cells from confluent monolayers were incubated for 10 minutes at 4°C in lysis buffer, cell lysates were cleared by centrifugation, and protein concentration was determined by using the Bio-Rad protein assay kit (Bio-Rad, Hercules, CA, USA). Due to the reduced amount of cells from each donor, in some experimentscells from different patients were pooled after treatments and analysed as a single experimental point.

**Real-time PCR and ELISA**

Ribonucleic acid was extracted from total cellular lysate and reverse transcribed using a commercially available kit (from Qiagen and Promega, respectively). RT-PCR analysis was performed using the iCycler™ apparatus (Bio-Rad) and the iQ™ SYBR® Green Supermix (Bio-Rad) under conditions recommended by the supplier. AAT primers, designed using the Primer3 software application, were sense: 5'- CCTATGATGAAGCGTTTAGG; antisense: 5'-TATCGTGGGTGAGTTCATTT. AAT expression levels were normalized to β-actin by the Q-gene software application [40].

Sandwich ELISA for total AAT was carried out in Costar® half-area plates (Corning Inc., Corning, NY, USA), which were coated by overnight exposure to polyclonal rabbit anti-human AAT antibody (against total AAT; DakoCytomation Ltd) diluted 1:500 in coating buffer (Carbonate-Bicarbonate Buffer; Sigma). After blocking of nonspecific binding sites, 50 υl of 10 × concentrated cell media or 10 μg of cell lysates were added to each well for 1 hour at 37°C. A standard curve (ranging from 100–1.6 ng/ml) was prepared using commercially available purified human AAT (Gentaur, Kampenhout, Belgium), and added to each assay. A horseradish peroxidase(HRP)-conjugated polyclonal goat anti-human AAT antibody (Abcam®, Cambridge, MA, USA) was added to the wells followed by the substrate, 1-Step Ultra TMB-Elisa (Thermo Scientific Inc., Rockford, IL, USA). The absorbance was estimated at 450 nm in an ELISA reader. Results are given as pg or ng of AAT per ml of culture medium or as pg of AAT per µg of protein.

Sandwich ELISA for polymeric AAT was performed as described [19, 26] with minimal modifications. Briefly, Costar plates were coated and blocked as described above. Wells were then incubated with 50 ul of concentrated media or 20 ug of cell lysates for 2 hours at 37°C. Preliminary experiments were also performed to assess that cell media concentration do not induce by itself Z-AAT polymer formation. Using culture media from 16HBE cells expressing Z-AAT we observed that results obtained from the analysis of un-concentrated media were consistently superimposable to the ones obtained from the correspondent volume of 10x concentrated media (data not shown).

Subsequently, the ATZ11 diluted 1:50 was added, followed by a HRP-conjugated antimouse antibody (GE Healthcare, Little Chalfont, Buckinghamshire, UK). After the addition of the substrate (1-Step Ultra TMB-Elisa; Thermo Scientific Inc.), the optical density was measured at 450 nm using an ELISA reader. Results are given as absorbance measured at 450 nm. All samples were measured in triplicate**.**
